# The World of Stable Ribonucleoproteins and Its Mapping With Grad-Seq and Related Approaches

**DOI:** 10.3389/fmolb.2021.661448

**Published:** 2021-04-07

**Authors:** Milan Gerovac, Jörg Vogel, Alexandre Smirnov

**Affiliations:** ^1^Institute of Molecular Infection Biology (IMIB), University of Würzburg, Würzburg, Germany; ^2^Helmholtz Institute for RNA-based Infection Research (HIRI), Helmholtz Centre for Infection Research (HZI), Würzburg, Germany; ^3^UMR 7156—Génétique Moléculaire, Génomique, Microbiologie (GMGM), University of Strasbourg, CNRS, Strasbourg, France; ^4^University of Strasbourg Institute for Advanced Study (USIAS), Strasbourg, France

**Keywords:** ribonucleoprotein, complexomics, Grad-seq, RNA-binding protein, noncoding RNA, sRNA, ProQ, FinO domain

## Abstract

Macromolecular complexes of proteins and RNAs are essential building blocks of cells. These stable supramolecular particles can be viewed as minimal biochemical units whose structural organization, i.e., the way the RNA and the protein interact with each other, is directly linked to their biological function. Whether those are dynamic regulatory ribonucleoproteins (RNPs) or integrated molecular machines involved in gene expression, the comprehensive knowledge of these units is critical to our understanding of key molecular mechanisms and cell physiology phenomena. Such is the goal of diverse complexomic approaches and in particular of the recently developed gradient profiling by sequencing (Grad-seq). By separating cellular protein and RNA complexes on a density gradient and quantifying their distributions genome-wide by mass spectrometry and deep sequencing, Grad-seq charts global landscapes of native macromolecular assemblies. In this review, we propose a function-based ontology of stable RNPs and discuss how Grad-seq and related approaches transformed our perspective of bacterial and eukaryotic ribonucleoproteins by guiding the discovery of new RNA-binding proteins and unusual classes of noncoding RNAs. We highlight some methodological aspects and developments that permit to further boost the power of this technique and to look for exciting new biology in understudied and challenging biological models.

## Introduction

When about two decades ago high-throughput approaches in molecular biology became a reality, it all began with parts lists. Genomics shed light on the ensemble of an organism’s genes and provided the first idea about what proteins and noncoding RNAs can in principle be there (Land et al., 2015; Encode Project Consortium et al., 2020). Transcriptomics identified and quantified various kinds of RNAs present in the cell under specific conditions (Wang et al., 2009; Lowe et al., 2017; Hör et al., 2018), and proteomics and metabolomics did the same for proteins and small molecules (Baran et al., 2009; Larance and Lamond, 2015; Omenn et al., 2016). But knowing the pieces does not yet mean playing chess. However essential such catalogs are, understanding what exactly every part does in the cell for a long time required one-by-one characterization, painstaking analysis of interactions with other parts, and rationalization of the ensuing biological effects.

Expectedly, next-generation high-throughput approaches, doing the same in a massively parallel way, soon emerged. Genome-wide functional screens were greatly facilitated by modern mutagenesis tools based on random transposon insertion and CRISPR-mediated gene disruption, silencing, or activation (Ford et al., 2019; Cain et al., 2020; Jiang et al., 2020). They now permit to simultaneously assess the importance of thousands of individual genes, including essential ones, under desired conditions (Langridge et al., 2009; Gilbert et al., 2014; Wang et al., 2014, 2015; Shalem et al., 2015; Peters et al., 2016). Orthogonally, high-throughput phenotyping enables the analysis of genotypes of interest against a wide palette of different conditions, providing insights into the cellular pathways the corresponding genes contribute to Nichols et al. (2011); Kritikos et al. (2017). The molecular mechanisms behind these phenotypes can be attained with interactomic approaches.

Over the last decade, many techniques have been developed to identify partners of specific RNAs and RNA-binding proteins (RBPs) with mass spectrometry-based proteomics and RNA-seq, respectively (Saliba et al., 2017; Giambruno et al., 2018; Lin and Miles, 2019). Among the latter, crosslinking approaches based on the covalent fixation of direct and often transitory RNA–protein associations, such as CLIP-seq, enjoy the widest popularity (Andresen and Holmqvist, 2018; Lee and Ule, 2018; Tree et al., 2018). The wealth of data obtained with these methods connected once-isolated proteins and RNAs into an intricate genome-wide web of interactions, delivering key information for understanding complex phenotypes (Quattrone and Dassi, 2019; Van Nostrand et al., 2020). At the same time, many questions regarding the biological meaning of these associations arose. Which of them take place simultaneously (e.g., on the same transcript) and which are mutually exclusive? Which binding events are just casual handshakes and which represent stable, persistent associations of macromolecules? Do these interactions occur in a simple, one-to-one manner or do they involve more elaborate, multi-subunit complexes? What is the actual diversity of such assemblies? Over the last years, the awareness increased that the virtual edges in RNA-protein interaction networks need to be converted into something more tangible and solid, endowed with clearly defined physical meaning. The time has come to compile the next-level parts list—that of ribonucleoprotein complexes (RNPs).

## What Is a Stable RNP?

RNA–protein associations are commonly subdivided in transient and stable. They differ not only in physicochemical properties but also in biologically relevant modes of action. Transiently interacting macromolecules, epitomized by enzyme–substrate complexes, do the best in fugitive encounters: this keeps the enzyme available for further rounds of reaction while leaving the product to go its way (consider RNases or RNA modification factors as examples). In contrast, as we will see below, stable RNPs can only carry their functions out if the partnership between the RNA and the protein persists for a relatively long time. This long-lasting association is a foundation for quite a peculiar kind of molecular behavior which is more characteristic of biological, as opposed to purely chemical, systems. It creates prerequisites for such biologically important properties as structuration, information flow, state maintenance, and switching. Stable complexes are also naturally more amenable to study, and consequently, we know much more about this group of RNPs. The present review will essentially focus on stable RNPs and the methods to characterize them on a global scale. Transient RNA–protein associations, e.g., most enzyme–substrate complexes, temporary low-affinity interactions between RNA chaperones and their clients, and other weak binding events, fall beyond its scope.

What does it take for an RNP to be called stable? Any would-be stable complex must meet at least one of the following requirements: (i) it must have a relatively low dissociation constant, at least in the sub-micromolar range (thermodynamic stability) and/or (ii) its off-rate must be fairly slow, typically <10^–4^ s^–1^ (kinetic stability). We refer the reader to the following publications for examples of these parameters among some well-studied RNPs (Yang et al., 2013; Nithin et al., 2019; Licatalosi et al., 2020). It is also instrumental to consider two operational definitions of stability: (i) the “*in vivo* stability,” when the complex is long-lived within the dynamic cell milieu teeming with thousands of other molecules (which may in various ways influence its integrity), and (ii) the “*in vitro* stability,” when the complex only needs to be intrinsically robust in (effective) isolation from other cellular components (Helder et al., 2016; Gehring et al., 2017). This distinction is practically very important because it means that we can analyze not only genuinely strong complexes, such as housekeeping RNPs, but also many other, normally dynamic ones which happen to be kinetically trapped, e.g., by dilution into cold buffer upon cell lysis, preventing them from remodeling, disassembly, or degradation (Licatalosi et al., 2020).

**FIGURE 1:**
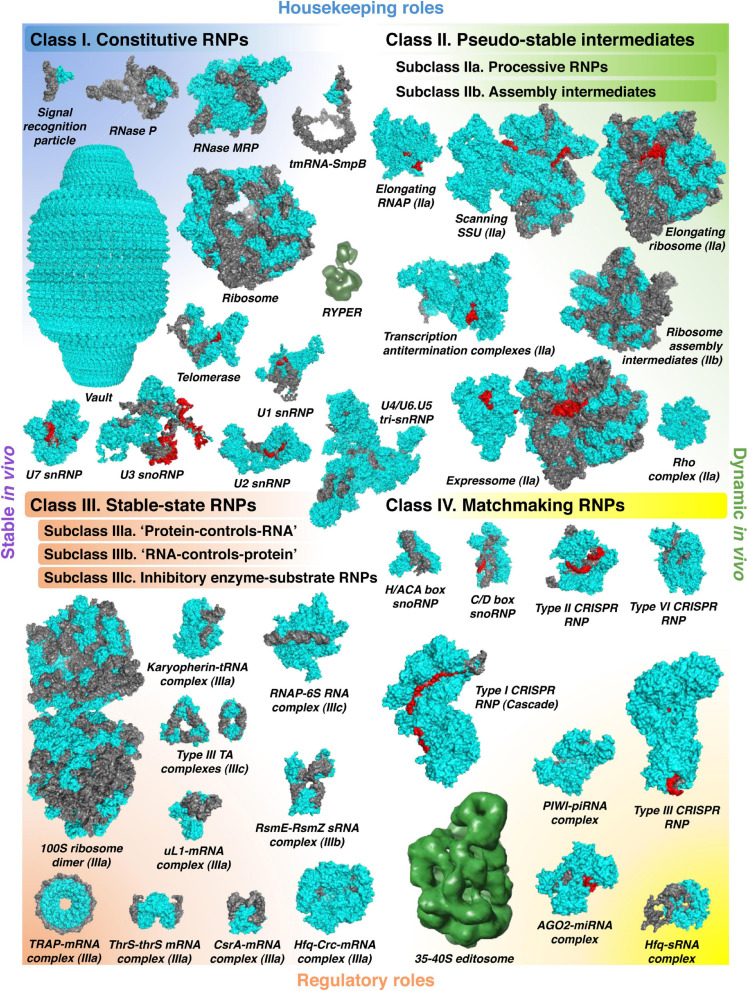
The world of stable RNPs. The wide diversity of stable RNPs existing in the cell can be presented as a continuum of assemblies with various biochemical and biological properties, roughly partitioned in four distinct classes. Constitutive RNPs (*class I*) are stable, permanent RNA–protein complexes, mostly with housekeeping roles. *Class II* comprises dynamic “on-pathway” intermediates, which can be artificially stabilized if deprived of the necessary building blocks. They are subdivided in the active complexes formed by translocating processive enzymes (*subclass IIa*) and the large RNP assembly intermediates (*subclass IIb*). Stable-state RNPs (*class III*) rely on strong facultative RNA–protein interactions where one of the partners influences the stability, the activity, or the localization of the other. In the *subclass IIIa*, RBPs regulate their RNA ligands by simple binding (often accompanied by the recruitment of other *trans*-acting factors) or by occlusion of other binding sites. In the *subclass IIIb*, on the contrary, RNAs regulate their protein partners, often by affecting their activity or localization. Unusually long-lived enzyme–substrate complexes, where the RNA substrate inhibits and traps the protein or RNP enzyme, are separated in the distinct *subclass IIIc*. Finally, the versatile RNPs, in which the RNA moiety anneals, with the help of its protein partner, to target nucleic acids, form the *class IV*. This group mainly includes regulatory RNPs but also several types of specialized housekeeping complexes. All classes are illustrated with examples whose structures have been solved (PDB codes: class I—1HQ1, 3Q1Q, 7C79, 3IYQ, 4V60, 7K00, 6D6V, 6G90, 6V4X, 6LQV, and 5GAN; class II—6RH3, 6YAM, 6WD0, 6TQO, 6TNN, 6ZTN, and 6DUQ; class III—6H58, 3ICQ, 5VT0, 4RMO, 2XDD, 2MF1, 2HW8, 1GTF, 1KOG, 2JPP, and 6O1M; class IV—2HVY, 4BY9, 5H9F, 4OO8, 6VRC, 5GUH, 6IFU, 4W5Q, and 4V2S). Proteins are shown in cyan, RNAs in gray, and their base-paired substrates or templates in red (except for RYPER and the 35–40S editosome, for which only low-resolution density maps are available: EMD-5389, EMD-1594; they do not permit to confidently distinguish the RNA and protein components).

Before discussing how this basic physicochemical principle can be converted into working methodology, we will provide an overview of diverse kinds of stable RNPs and explain why they deserve most thorough investigation. Without aspiring to a catch-all classification, we can sort the majority of known stable RNPs in four major classes, based on their biological properties and/or activities and the nature of the interaction between the RNA and the protein components (Figure 1). In fact, the distinction between these classes is sometimes blurred, and some complexes can be well assigned to more than one category. Therefore, it is more appropriate to speak about a continuum of stable RNPs, with four poles typified by relatively “pure” examples and many intermediate cases in between. The main purpose of this classification is to show what kind of stable RNPs fall in the scope of the existing global biochemical approaches, in particular, the complexomic methods described in the second part of this review. We will primarily use examples from bacterial RNA biology, but some particularly interesting cases from eukaryotes will be invoked as well to highlight the general bearing of the proposed ontology.

## Ontology of Stable RNPs

### Class I: Constitutive RNPs

Constitutive RNPs are highly organized, permanent RNA-protein associations where one or both partners provide structural and functional support to each other. Much of our current knowledge on the molecular interplay between the RNA and the protein components of ribonucleoproteins comes from the research on constitutive RNPs (Martin and Reiter, 2017). This interplay is remarkably variable. In ribonucleoprotein RNases P, the RNA subunit performs pre-tRNA cleavage, whereas the associated protein(s) assist in its folding and substrate recognition (Guerrier-Takada et al., 1983; Reiter et al., 2010; Wan et al., 2019a; Lan et al., 2020). On the contrary, in the signal recognition particle, the RNA bears the burden of scaffolding and promotes the interaction with the SRP receptor, while a protein subunit works as the enzymatic moiety performing GTP hydrolysis (Peluso et al., 2000). In the ribosome, rRNA usually excels in both duties, serving as the skeleton and the catalytic heart of this giant complex, whereas proteins are relegated to secondary roles (Ban et al., 2000; Muth et al., 2000; Nissen et al., 2000; Routh and Sankaranarayanan, 2017). However, mitochondrial ribosomes often deviate from this rule: in the extreme case of trypanosomatids, the mitochondrial rRNA has suffered such profound structural erosion that the scaffolding task almost entirely falls to the hypertrophied protein shell (Ramrath et al., 2018; Soufari et al., 2020).

Behind all these variations, there is a common architectural theme followed by all constitutive RNPs: both RNA and protein make up a unique and inseparable whole and thereby contribute to the same, shared molecular function. The components of constitutive RNPs are normally bound in an obligate association with each other and only occasionally work outside this context (Pelava et al., 2016; Meyer, 2018; Nakagawa et al., 2018). They are fully hardwired pieces of the cell circuitry. The extreme degree of structural and functional integration earned the most sophisticated of them the qualification of “molecular machines,” and the current physical description of their workings supports this metaphor (Valle et al., 2003; Korostelev et al., 2008; Wilkinson et al., 2020).

As the above-cited examples show, most known constitutive RNPs are housekeeping. The molecular functions of others, like the imposing 13-MDa vault, remain elusive (Tanaka et al., 2009). But can constitutive ribonucleoproteins play regulatory roles? A potential step in this direction is Ro60-Y RNA complexes (Boccitto and Wolin, 2019). In *Deinococcus radiodurans*, the Ro60-like protein Rsr is tethered via Y RNA to the major bacterial exoribonuclease PNPase and seems to change its substrate specificity by promoting the degradation of structured RNAs (Chen et al., 2013; Sim and Wolin, 2018). This so-called RYPER complex is assembled in response to certain stresses, such as UV irradiation or prolonged stationary phase, and in *Salmonella enterica*, expression of Y-like RNAs, engaged in a similar kind of RNPs, appears to be confined to certain infection stages (Chen et al., 2013; Westermann et al., 2016), suggesting that they play condition-specific roles in RNA metabolism.

Even more striking cases of regulatory constitutive RNPs are found in the group of scaffolding lncRNAs (Chujo et al., 2016; Smith et al., 2020a). To cite just one telling example, eukaryotic paraspeckles are organized around an architectural lncRNA, NEAT1, stably associated with several RBPs, such as NONO, SFPQ, and FUS (Yamazaki et al., 2019). This core RNP is thought to represent a structurally heterogeneous latticework of RNA-RNA and RNA-protein interactions that nucleate the coalescence of the outer shell components, giving rise to a phase-separated condensate (Fox et al., 2018; Yamazaki et al., 2018). Paraspeckles have been linked with gene expression regulation at various levels, not least by localizing or sequestering specific proteins and transcripts (Chujo et al., 2016; Nakagawa et al., 2018). Interestingly, several families of large and ornately structured noncoding RNAs have been detected in bacteria (Weinberg et al., 2009, 2017; Harris and Breaker, 2018). One of them, OLE, is an abundant, stable transcript, forming membrane-associated RNPs in many Firmicutes to protect bacteria from cold and envelop stress (Puerta-Fernandez et al., 2006; Block et al., 2011; Wallace et al., 2012; Harris et al., 2019; Widner et al., 2020). However, the molecular mechanisms and the biological functions of this and other bacterial lncRNAs still need to be established.

### Class II: Pseudo-Stable Intermediates

#### Subclass IIa: Processive Enzyme–Substrate Complexes

As already said, enzyme–substrate complexes are usually not stable. There are, however, a couple of interesting exceptions. Processive enzymes perform multiple rounds of catalysis without releasing the substrate into solution, like it happens with the elongating RNA polymerase (RNAP), the scanning eukaryotic translation initiation complex, the elongating ribosome, or the translocating transcription termination factor Rho (Hinnebusch, 2011; Gocheva et al., 2015; Kriner et al., 2016; Brito Querido et al., 2020). Of course, such complexes are “stable” only by first approximation, at the compositional level. In reality, they represent an ensemble of discrete short-lived elongation states. Below we will see how this ambivalence can be exploited to glean insight into the mechanics of key gene expression processes.

Translation of an average bacterial ORF, specifying a protein of ∼50 kDa, takes ∼25 s; and during this time, the mRNA and the ribosome are bound to stay together (Johnson et al., 2020). This does not seem particularly long, meaning that processive RNPs are not stable by “*in vivo*” standards. But this offers a time window generous enough for the experimenter to intervene and leverage their hidden “*in vitro* stability”. Indeed, many processive RNPs have evolved to firmly hold their template and/or the elongated substrate until a termination signal occurs and enables their dissociation. Yet, elongation depends on physiological temperatures and the regular supply of building blocks (nucleoside triphosphates for RNAP, aminoacyl-tRNAs for the ribosome). If one rapidly cools down the system and dilutes out the building blocks, the bereft processive RNP will stall dead-still and become analyzable. This principle underlies the ribosome profiling, which permits to follow the translational dynamics of all mRNAs in the cell simultaneously (Ingolia, 2016). The same physicochemical basis underlies the sequencing of nascent transcripts (NET-Seq and RNET-seq) and various versions of the transcription run-on assay to create a global snapshot of RNAP activity across the genome (Imashimizu et al., 2015; Jordán-Pla et al., 2019). In either case, the positions of RNA/DNA footprints or toeprints report on the location of the elongating processive RNP on the template.

As seen from these examples, pseudo-stable processive RNPs are deeply involved with core gene expression processes. A singular example of such complexes is the bacterial expressome that physically couples transcription and translation by joining an elongating RNAP and a ribosome via NusG and NusA proteins (O’Reilly et al., 2020; Wang et al., 2020a; Webster et al., 2020). Formation of the expressome and the associated effects on transcription elongation (e.g., antitermination and “pushing” of backtracked RNAP) depend on the relative speed of RNAP and the pioneering ribosome and take place only in those bacteria where the velocities of both molecular machines are properly matched (Burmann et al., 2010; Proshkin et al., 2010; Johnson et al., 2020; Stevenson-Jones et al., 2020).

The inherently variable elongation speed, open to modulation by sequence features and *trans*-acting factors, creates a fertile ground for regulation. Indeed, genome-wide studies revealed that both RNAP and ribosomes frequently pause, this pausing is often contingent on a specific molecular context or environmental conditions and may profoundly affect the cellular state (Chan et al., 2012; Shalgi et al., 2013; Subramaniam et al., 2014; Richter and Coller, 2015; Choi et al., 2018; Neugebauer, 2019; Yakhnin et al., 2020). The differential speed of RNA polymerase II can influence the pattern of cotranscriptional alternative splicing and thereby effect isoform switching (Muñoz et al., 2009; Ip et al., 2011). In mammalian mitochondria, regulation of RNAP processivity by the transcription elongation factor TEFM is of paramount importance, as it ultimately decides between the whole-genome transcription and the RNA-primed replication of the organellar DNA (Agaronyan et al., 2015; Hillen et al., 2017). Furthermore, the elongation of mitochondria-synthesized polypeptides is moderated to accurately match the incorporation of incoming nuclear-encoded subunits as respiratory complexes are being assembled (Couvillion et al., 2016; Richter-Dennerlein et al., 2016; Wang et al., 2020b).

Bacteria evolved elaborate regulatory strategies operating at the level of transcription elongation, including riboswitches, protein-dependent attenuators, Rho-mediated termination, and a variety of processive antitermination and pausing mechanisms that often involve NusG family proteins (Yakhnin et al., 2006, 2020; Said et al., 2017; Goodson and Winkler, 2018; Kang et al., 2018; Turnbough, 2019; Huang et al., 2020). Additionally, some Hfq-dependent sRNAs, traditionally perceived as posttranscriptional regulators, have been implicated in *E. coli* RNAP elongation by countering the Rho-dependent termination of the target mRNA (Sedlyarova et al., 2016). One of the most intricate examples of using processive RNPs in regulatory decision-making is leader peptide attenuators. These widespread switch elements elegantly subordinate the transcription of downstream genes to translation elongation on a short upstream ORF (Turnbough, 2019). The two processes are conditionally coupled, depending on the availability of the “sensed” nucleoside triphosphates (Turnbough et al., 1983; Bonekamp et al., 1984; Roland et al., 1988), aminoacyl-tRNAs (Artz and Broach, 1975; Lee and Yanofsky, 1977; Oxender et al., 1979; Johnston et al., 1980), or, in one described case, elongation factor P (Nam et al., 2016). The relative speed and eventual pausing of either machinery determine whether RNAP transcribes all the way down the operon or terminates prematurely (Turnbough, 2019).

Bacteria and eukaryotes also use purely translational regulatory mechanisms relying on the conditional slowdown—up to a complete arrest—of the elongating ribosome (Wilson et al., 2016; Choi et al., 2018). These include the prototypic *secMA* system, controlling the expression of the protein translocase SecA (Nakatogawa and Ito, 2001; Mitra et al., 2006), and some regulated antibiotic resistance cassettes (Arenz et al., 2014).

#### Subclass IIb: Assembly Intermediates

A behavior conceptually reminiscent of that of processive RNPs is found in the assembly pathways of complex molecular machines. The ribosome biogenesis proceeds via a series of defined intermediates and is pulled forward by the sequential recruitment of ribosomal proteins and assembly factors, which serve as building blocks (Shajani et al., 2011; Klinge and Woolford, 2019). Assembly intermediates are naturally short-lived *in vivo*, representing lowly populated states of nascent RNPs. However, some of them exist long enough or become sufficiently abundant (e.g., in exponentially growing bacteria) to enable their capture in the same way as described above, i.e., by snap cooling, dilution, and separation from other cellular components (Chen and Williamson, 2013). This is often achieved by pulling down the intermediate of interest via a unique marker protein or an RNA region (Gupta and Culver, 2014; Wu et al., 2016; Zhang et al., 2016; Barandun et al., 2017; Chaker-Margot et al., 2017; Chen et al., 2017b; Cheng et al., 2017, 2019, 2020; Heuer et al., 2017; Hunziker et al., 2019). But the most decisive—and impressive—implementation of this “*in vitro* stabilization” workaround has been attained in cryo-EM studies relying on advanced particle classification from heterogeneous samples. These works brought about unique data on the architecture of assembly intermediates and biogenesis factors for ribosomes and spliceosomes—and this without perturbing the natural assembly pathways (Sashital et al., 2014; Brown et al., 2017; Ameismeier et al., 2018; Wan et al., 2019b; Itoh et al., 2020; Jaskolowski et al., 2020; Soufari et al., 2020). Alternatively, intermediates can be enriched by “starving” the assembly for one of the building blocks. This approach is widely adopted as it permits to generate higher amounts of analyzable complexes, which is particularly instrumental in studying very short-lived ribosome and spliceosome assembly stages (Li et al., 2013; Jomaa et al., 2014; Davis et al., 2016; Ni et al., 2016; Sun et al., 2017; Zeng et al., 2018; Razi et al., 2019; Seffouh et al., 2019; Du et al., 2020; Rabuck-Gibbons et al., 2020; Rai et al., 2021). However, this comes at a risk of altering the native assembly route and accumulating off-pathway, dead-end particles.

### Class III: Stable-State RNPs

Stable-state RNPs form the most numerous and actively studied RNP class. Like class I RNPs, they rely on strong RNA-protein interactions. Contrary to the former group, however, these interactions are not obligate and rather represent one of two (or sometimes more) distinct alternative states, each associated with a (dramatically) different outcome for the partners. Usually very long-lived *in vitro*, such complexes may be dynamic *in vivo* as other, competing molecules eventually drive one of the partners out and thereby switch the state of the system. Such properties open endless possibilities for gene expression control. Depending on which partner suffers (or enjoys) the consequences of such an interaction and whether it involves simple RNA binding or an enzymatic event, class III can be subdivided in “protein–controls–RNA” or “RNA–controls–protein”-type RNPs and inhibitory enzyme–substrate complexes.

#### Subclass IIIa: “Protein–Controls–RNA”-Type RNPs

This is likely the most widespread kind of stable-state RNPs: a protein binds a transcript and influences its stability, localization or, in the case of mRNAs, translation. In eukaryotes, most of such regulation converges at the level of the extensive 3′-UTR harboring recognition sites for multiple RBPs and miRNAs (Mayya and Duchaine, 2019). This enables combinatorial control of prodigious complexity where diverse *trans*-acting factors boost, mitigate, or override the effects of each other, thereby achieving highly nuanced outcomes (Iadevaia and Gerber, 2015; Dassi, 2017). Bacteria also set great store by regulating their mRNAs but do this usually via the 5′-UTR, near the translation start site, where most bacterial RBPs and sRNAs bind (Meyer, 2017; Holmqvist and Vogel, 2018b). The paradigm of such mechanisms is the broadly conserved protein CsrA that strongly binds to ANGGA motifs so frequent in Shine-Dalgarno sequences (Schubert et al., 2007; Holmqvist et al., 2016; Potts et al., 2017; Romeo and Babitzke, 2018). This preference makes CsrA family proteins potent translational repressors whose high-affinity binding can only be overcome by a dedicated protein antagonist, such as FliW or CesT, or a special group of “sponge” RNAs, which will be described in the next section (Mukherjee et al., 2011, 2016; Altegoer et al., 2016; Dugar et al., 2016; Katsowich et al., 2017; Sowa et al., 2017; Ye et al., 2018; Oshiro et al., 2019). In rare cases, CsrA upregulates the expression of its target through mRNA stabilization or translational activation (Yakhnin et al., 2013; Renda et al., 2020).

A beautiful example of translational feedback control is the *Bacillus subtilis* undecameric RBP TRAP that, under tryptophan-replete conditions, binds to the 5′-UTR of tryptophan-related mRNAs and prevents their translation (Merino et al., 1995; Du et al., 1997; Du and Babitzke, 1998; Babitzke, 2004; Yakhnin et al., 2004). Another widespread mechanism of translational repression is the autoregulation of ribosomal protein operons: some r-proteins interact with a specific region in the 5′-UTR of their own mRNA that mimics their native binding site within the ribosome, thereby inhibiting their own translation as well as the production of other r-proteins encoded on the same polycistronic message (Meyer, 2018). A similar molecular mimicry case is the autoregulation of the *E. coli* threonyl-tRNA synthetase, which recognizes a tRNA^*Thr*^-like element in its own 5′-UTR (Romby et al., 1996; Torres-Larios et al., 2002). Interestingly, the RNA chaperone Hfq, more known as an sRNA cofactor (see *Class IV*), can itself serve as a translational inhibitor of certain mRNAs, including its own (Večerek et al., 2005; Desnoyers and Massé, 2012; Chen and Gottesman, 2017; Azam and Vanderpool, 2018; Morita and Aiba, 2019). In *Pseudomonas*, Hfq joins forces with another global regulator, Crc, to shut down the translation of numerous mRNAs (Moreno et al., 2015; Sonnleitner et al., 2018; Pei et al., 2019). Recent data indicate that RBP association with target mRNAs in bacteria may occur cotranscriptionally and, similar to eukaryotes, support combinatorial regulatory modes (Kambara et al., 2018; Gebhardt et al., 2020; Melamed et al., 2020).

The examples just quoted give class IIIa RNPs a uniquely regulatory flair. However, their housekeeping roles are not to be underestimated: in eukaryotes, such complexes punctuate the ages of the normal life cycle of many cellular transcripts (Müller-McNicoll and Neugebauer, 2013). Numerous RBPs govern the intracellular localization of transcripts—a feature present not only in morphologically complex eukaryotic but also in simpler bacterial cells (Nevo-Dinur et al., 2011; Moffitt et al., 2016; Eliscovich and Singer, 2017; Béthune et al., 2019; Mahbub et al., 2020). The cap-binding complex, poly(A)-binding protein, and the associated factors serve as tokens of stability and translatability of eukaryotic mRNAs (Müller-McNicoll and Neugebauer, 2013; Rissland, 2017). It is likely that a somewhat analogous role is played in bacteria by Hfq, which stably associates with the intrinsic terminators of both coding and noncoding RNAs, protecting them from 3′-to-5′ exoribonucleases (Sauer and Weichenrieder, 2011; Holmqvist et al., 2016).

In a few special cases, regulatory proteins form stable-state complexes with entire class I RNPs, like it happens with the ribosome-associated inhibitor RaiA, that stabilizes bacterial ribosomes in an inactive 70S form, or the dormancy factors HPF and RMF, which induce ribosome dimerization into inert 100S particles under stress, stationary phase, and sometimes even normal growth conditions (Kato et al., 2010; Polikanov et al., 2012; Matzov et al., 2017). Similar mechanisms have been described in eukaryotes (Ben-Shem et al., 2011; Brown et al., 2018; Barandun et al., 2019). There exist also examples of regulatory RBPs that inhibit ribosomes translating specific mRNAs (Darnell et al., 2011; Chen et al., 2014).

#### Subclass IIIb: “RNA–Controls–Protein”-Type RNPs

In this scenario, opposite to the previous one, the RNA rules over its protein partner. This rule is not necessarily a repressive one: for example, many lncRNAs simply recruit specific proteins to the sites where their activity is required (Kopp and Mendell, 2018). Nevertheless, it is more common to showcase “RNA–controls–protein” complexes with so-called sequestration mechanisms. In classical regulation by sequestration, the decoy RNA lures the cognate RBP into a tight but unproductive association, effectively debarring the protein from acting on its *bona fide* RNA targets. Although protein sequestration by RNA is found in eukaryotes too (Kino et al., 2010; Lee et al., 2016; Egloff et al., 2018), the finest examples thereof arguably come from bacterial RNA biology.

The *E. coli* small RNA decoy GlmY mimics its mate sRNA GlmZ in all respects but the presence of the base-pairing region employed by GlmZ to activate the translation of the *glmS* mRNA (Reichenbach et al., 2008; Urban and Vogel, 2008). GlmS makes glucosamine-6-phosphate (GlcN6P) required for the bacterial cell wall synthesis (Milewski, 2002). When GlcN6P is abundant, a regulatory RBP, RapZ, captures GlmZ and delivers it to RNase E for cleavage; this prevents unnecessary GlmS production (Göpel et al., 2013, 2016; Durica-Mitic et al., 2020). But when GlcN6P is scarce, GlmY jumps in and titers RapZ out by chaining it in stable—and completely inert—class IIIb RNPs (Gonzalez et al., 2017a; Khan et al., 2020). GlmZ thereby escapes degradation and activates *glmS* translation (Göpel et al., 2013).

RapZ is a very specific protein and its sequestration has only local regulatory consequences. How much more far-reaching could be the effect of detaining a globally acting RBP! This is exactly what happens to the *Pseudomonas* Hfq protein sponged by the abundant CrcZ sRNA (Sonnleitner and Bläsi, 2014; Pusic et al., 2016; Sonnleitner et al., 2017). The currently best-understood case of such control, from both functional and structural perspectives, is provided by the widespread noncoding RNAs that sequester members of the already mentioned CsrA/Rsm protein family (Babitzke and Romeo, 2007). These decoy sRNAs are basically a concatenation of numerous GGA motifs specifically recognized by CsrA-like proteins. Most of these potential binding sites are low (micromolar) affinity, when considered in isolation. But together they show high cooperativity, so that the initial fleeting and seemingly innocuous encounter with the target protein turns the decoy RNA into a molecular “black hole” avidly absorbing multiple CsrA dimers (Duss et al., 2014). As a result, CsrA is no longer available for repressing its mRNA targets, and an entire large regulon gets activated (Romeo and Babitzke, 2018).

#### Subclass IIIc: Inhibitory Enzyme–Substrate RNPs

We have seen above that enzyme–substrate complexes sometimes conceal remarkable *in vitro* stability, which can be accessed by relatively simple experimental means. In contrast to those class IIa RNPs, inhibitory enzyme–substrate complexes are long-lived *in vivo*. They arise as a special kind of stable-state RNPs formed in an unusual, indeed conspicuous, manner. An enzyme, which is fully active on its regular RNA substrates, eventually encounters an unusual one, which it cannot process normally, and both partners freeze in a stable complex. The simplest realization of this scenario is found in type III toxin–antitoxin systems used to abort phage infection or stabilize plasmids (Goeders et al., 2016). Here an endoribonuclease toxin is physiologically inactivated by the cognate pseudoknotted RNA antitoxin, with which it forms a highly stable, closed RNP (Blower et al., 2011; Samson et al., 2013; Short et al., 2013; Rao et al., 2015). This inhibitory complex forms via a processing reaction performed by the toxin on the antitoxin precursor: the enzyme inadvertently snares itself as it cleaves the “poisoned” substrate. This prevents other RNAs from accessing the toxin, rendering the latter perfectly harmless for the host, at least under normal circumstances (Short et al., 2013, 2018).

In another mechanism broadly conserved among bacteria, the housekeeping form of RNAP is fooled into binding to 6S RNA that structurally imitates a molten DNA duplex (Wassarman, 2018). The resulting complex is extremely stable and effectively sequesters RNAP from σ^70^-dependent promoters, tilting the balance in favor of alternative σ-factors, such as σ^*S*^, insensitive to 6S RNA (Wassarman and Storz, 2000; Gildehaus et al., 2007; Chen et al., 2017a). In this stable state (typically observed when bacteria enter the stationary phase), transcription undergoes global remodeling (Cavanagh et al., 2008). However, when new resources permit to resume growth, the system must be reset. RNAP cannot simply “spit out” the 6S RNA and is forced to disentangle itself from the problematic substrate by using it as a template to synthesize a short product RNA (pRNA) (Wassarman and Saecker, 2006; Gildehaus et al., 2007). The conformational change operating during this weird transcriptional act forces the 6S RNA-pRNA duplex out (Wurm et al., 2010; Beckmann et al., 2012; Chen et al., 2017a). An analogous system has been reported in mammalian cells: the SINE-encoded B2 RNA competitively inhibits RNA polymerase II and, just like 6S RNA, exploits self-templated transcription as a release mechanism (Espinoza et al., 2004; Yakovchuk et al., 2009; Wagner et al., 2013).

Inhibitory enzyme–substrate complexes may form accidentally, e.g., when RNAP encounters a DNA damage site or a ribosome translates an aberrant mRNA. Their resolution is ensured by dedicated cellular mechanisms, including the Mfd-mediated disassembly of the transcription elongation complex (Selby and Sancar, 1993; Shi et al., 2020; Kang et al., 2021) and the ribosome rescue by trans-translation and no-stop/no-go-mediated pathways (Keiler, 2015; Simms et al., 2017).

### Class IV: Matchmaking RNPs

Matchmaking RNPs can be counted among the most advanced evolutionary inventions in the domain of nucleic acid recognition. Their tremendous success in all three branches of life is largely due to their unique labor division scheme, radically different from what RBPs typically do (Liu et al., 2020). The latter recognize and act upon their targets with the help of elaborate binding sites that are intrinsically predisposed to accept more-or-less well-defined short RNA sequences embedded in a particular kind of 3D structure (Jankowsky and Harris, 2015). With some exceptions (Hall, 2016), the RNA recognition code used by RBPs is notoriously complex and not in the least universal. By contrast, within matchmaking RNPs, the task of target discrimination is entrusted to an RNA moiety that relies on a simple and universal set of base-pairing rules and can operate on longer sequences and not always in the best structural context. The protein subunit of such RNPs assumes the role of protector and presenter, ensuring that the RNA is displayed in a conformation optimal for rapid querying of potential targets; it also facilitates formation of a stable duplex, if the match is deemed satisfactory, and sometimes performs further molecular acts (Gorski et al., 2017).

Beside protein-based regulators, the class IV RNPs stand out as truly versatile RNA binders – and not only because of a more flexible target recognition strategy. Their key advantage is that they work as programmable devices where an RNA-“instruction” is plugged into the universal protein-“player” to chase and manipulate one specific kind of targets. The same “player,” however, can be reprogrammed with another RNA, which imparts a new specificity to hunt down different molecular preys. As a corollary, matchmaking RNPs, aptly dubbed “search engines” in a recent review (Dendooven and Luisi, 2017), can – via a huge number of possible guides – target practically any gene in the cell. Because such guiding RNA moieties are much easier to evolve than the equivalent number of target-specific RBPs, entire suites of new regulatory RNAs emerge around the same “player” protein, forming new functional classes within the noncoding transcriptome (Dutcher and Raghavan, 2018; Jose et al., 2019). The protein in its turn accedes to the status of “hub” and tremendously increases its regulatory reach and physiological importance (Vidal et al., 2011).

Because of the “target/programming” lingo, matchmaking RNPs are naturally associated with regulation. Indeed, in bacteria and eukaryotes special small RNA subtypes have been implicated in gene expression control. The widely conserved bacterial homohexameric Sm-like RNA chaperone Hfq interacts with over a hundred sRNAs, primarily engaging their intrinsic terminators (Holmqvist et al., 2016; Updegrove et al., 2016). This interaction is required for their stability and target recognition (Moll et al., 2003; Otaka et al., 2011; Ishikawa et al., 2012; Dimastrogiovanni et al., 2014). The latter is achieved with the help of the single-stranded base-pairing module, or seed sequence, typically situated on the 5′-end of sRNAs (Balbontin et al., 2010; Papenfort et al., 2010; Sauer et al., 2012; Fröhlich et al., 2013; Dimastrogiovanni et al., 2014). When the Hfq-sRNA complex encounters a true mRNA target, the protein catalyzes the duplex formation between the two transcripts (Santiago-Frangos and Woodson, 2018). Depending on the position of the duplex with respect to the translation initiation site, this RNA-RNA interaction may lead to translational inhibition, activation, or a change in the mRNA stability (Wagner and Romby, 2015). Although the sRNA-Hfq complexes are thermodynamically stable *in vitro* (with typical *K*_*d*_s in the low-nanomolar range), they show rapid exchange dynamics *in vivo*, where many different RNAs incessantly chase each other from the limited number Hfq hexamers (Wagner, 2013). At the systems level, this means that the population of Hfq-sRNPs is never the same and changes in function of the transcriptional profile which, in its turn, adapts to environmental conditions (Chao et al., 2012; Chihara et al., 2019). By this means, the Hfq-dependent regulon is constantly remodeled and stays flexible to provide rapid and adequate regulatory responses (Wagner and Romby, 2015). Very similar targeting mechanisms are employed in eukaryotes by Argonaute-associated small RNAs (Salomon et al., 2015; Sheu-Gruttadauria and MacRae, 2017).

Other types of matchmaking RNPs are involved in housekeeping processes. Probably the most ancient realization of the RNA-guided recognition principle can be found in the ribosome: bacterial 30S subunits typically find ORFs within “target” mRNAs with the help of the anti-Shine-Dalgarno sequence at the 3′-end of 16S rRNA (Shine and Dalgarno, 1974; Steitz and Jakes, 1975). Furthermore, the U1, U2, and U6 snRNPs (built around Sm-family protein oligomers akin to Hfq) exploit RNA-RNA base-pairing rules to locate the 5′-splice site and the branch point in pre-mRNAs (Papasaikas and Valcárcel, 2016). Other similar cases include U3 snoRNP, that chaperones critical pre-18S rRNA regions during SSU assembly (Barandun et al., 2018), and U7 snRNP (another Sm-ring complex), that guides the 3′-processing of histone mRNAs (Sun et al., 2020). However, in all these examples, guiding RNA moieties are stably integrated and cannot be exchanged to target different suites of transcripts. Much closer to the bespoke matchmakers described in the previous paragraph are snoRNPs and scaRNPs, that employ invariant protein scaffolds and different RNA guides to direct 2′-O-methylation or pseudouridylation at specific sites of archeal and eukaryotic rRNAs and snRNAs (Dupuis-Sandoval et al., 2015; Bohnsack and Sloan, 2018). Similarly, the RNA editosome edits several kinetoplast messengers in trypanosomatids using an ∼40-protein scaffold and ∼1,200 distinct guide RNAs (Göringer, 2012).

Special classes of matchmaking RNPs are employed as genome defense agents. For instance, siRNA-programmed AGO2 is used to combat viral infection in fungi, plants and invertebrates (Ding, 2010). PIWI-interacting RNAs repress transposons via a “ping-pong” cycle of base-pairing events in the germ line of most animals (Ozata et al., 2019). Finally, the large universe of CRISPR systems provides a number of fascinating mechanisms by which crRNA-guided endonucleases, either embedded in exuberantly complex RNPs or, on the contrary, stunningly simplistic, seek and destroy parasitic nucleic acids in prokaryotes (Mohanraju et al., 2016).

## Why a High-Throughput Approach to Stable RNPs?

Our knowledge of this mesmerizing diversity and mechanisms of stable RNPs is the fruit of decades of research relying on diverse biochemical, structural, and molecular biology techniques. While many of these methods continue to play a critical role by laying a solid ground for our understanding of these tiniest biological units, the next-generation, high-throughput approaches are knocking ever louder on the door. The impetus for their development is not only down-to-earth pragmatic (i.e., massively parallel RNP characterization). A comprehensive, genome-wide approach actually becomes a necessity when big, transversal questions are raised. One such question is ontological: what stable RNPs exist in the studied biological system under given conditions? Here the profiling nature of a high-throughput technique by far outperforms the serendipity of the traditional “by-chance” discovery. Another advantage of a “bird’s-eye” view on the cellular RNP ensemble is the access to systems-level information: what is the functional state of the cell? How are resources allocated among various RNP-based processes, such as transcription, RNA processing, translation, or degradation? How are the ongoing regulatory programs implemented on the level of persistent RNA-protein associations? Regarding the interactions themselves, genome-wide techniques provide unique biochemical data on the “*in vitro*” (affinity distribution, specificity) and “*in vivo*” (occupancy, competition, interconversion) parameters of RNPs (Campbell and Wickens, 2015; Jankowsky and Harris, 2015). And if one wishes to recast all this in a form of defined, tangible physical entities, as advocated here, a complexomic approach will be an obvious option.

## Profiling Cellular Complexes With Complexomic Techniques

**FIGURE 2:**
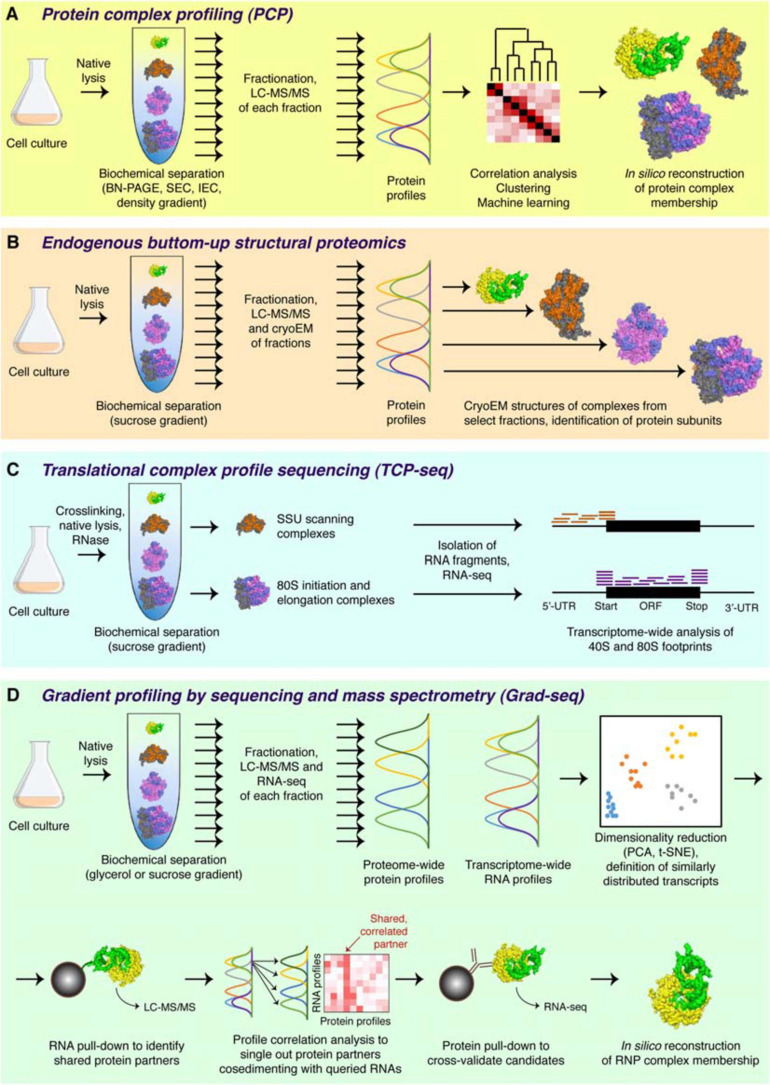
Major complexomic approaches. **(A)** In protein complex profiling (PCP), biological material is lysed under native conditions and resolved by one or several biochemical techniques (here velocity sedimentation in a density gradient). Each fraction is then analyzed by quantitative mass spectrometry, and protein profiles are cross-correlated to infer their involvement in the same or different complexes. **(B)** In endogenous bottom-up structural proteomics, the cell lysate is resolved on a sucrose gradient and profiled by mass spectrometry, like in PCP. In addition, individual fractions are subjected to cryoEM, followed by unsupervised, model-free 3D particle classification to determine structures of individual complexes and directly identify their protein constituents. **(C)** In the ribosome profiling family of approaches, represented here by TCP-seq, the lysate is chemically crosslinked, treated with an RNase to digest unprotected mRNA, and subjected to sucrose gradient centrifugation. Only the 40S and the 80S fractions are collected for subsequent analysis by RNA-seq to visualize the footprints of scanning 40S subunits or translating 80S ribosomes. **(D)** In Grad-seq, density gradient centrifugation is used to resolve RNPs up to the size of a monosome. Both the protein and the RNA components of each fraction are profiled by mass spectrometry and RNA-seq, respectively. The RNA profiles are clustered to identify cohorts of similarly behaving transcripts (likely forming the same kind of RNPs). Select members of each cohort are used as baits in pull-down assays to identify their protein partners. Then, the sedimentation profiles of the enriched proteins are correlated with those of the RNA baits, and the most consistent and recurrent candidates are cross-validated by RIP-seq or CLIP-seq. If the mutual interaction is confirmed, the RNP membership of the analyzed cohort can be considered established.

The logic of the complexomic approach is radically different from that of the traditional bait-prey interactomics (Smirnov et al., 2017a). In complexomics, there are no baits nor preys, no need to tag or catch molecules. In fact, this group of methods does not directly profile interactions; what it looks at is macromolecular complexes, their composition, and physical properties. In a typical complexomic experiment (Figure 2A), biological material (cultured cells or a tissue) is lysed and resolved by one or several biochemical techniques that partition complexes according to a certain physicochemical parameter (size, shape, charge, hydrophobicity etc.). Upon fractionation, the content of each fraction is analyzed quantitatively, and the profiles of individual macromolecules across the whole separation range are reconstructed computationally. Comparison of their distributions (“correlation profiling”), often in conjunction with the physical information gleaned from the separation principle applied, permits to assign macromolecules to distinct complexes, evaluate the composition of known assemblies, and even propose new ones.

Each step of this standard pipeline can be played differently: the last two decades witnessed a flurry of studies employing a wide diversity of complexomic approaches tailored to various biological systems and research questions. For example, Blue-Native (BN) PAGE is traditionally used in microbial and mitochondrial complexomics as it permits to analyze large (up to 30 MDa), soluble or membrane-embedded protein complexes, which cannot be satisfactorily resolved by other techniques (Schägger and Von Jagow, 1991; Strecker et al., 2010). In earlier 2D implementations, BN-PAGE gels were stained, and individual protein spots were identified by western blotting or mass spectrometry (Camacho-Carvajal et al., 2004; Farhoud et al., 2005; Pyndiah et al., 2007; Klodmann et al., 2011). Such an approach was naturally limited in scope as it was restricted to abundant proteins (or those for which specific antibodies were available). Therefore, it has been supplanted by 1D BN-PAGE with regular gel slicing and systematic LC-MS/MS analysis of each slice (Wessels et al., 2009). This extremely successful method enabled unbiased profiling of hundreds of proteins across one gel, assignment of molecular weights to the complexes they form, and the possibility to compare such profiles proteome-wide to infer protein complex memberships in many species (Helbig et al., 2009; Heide et al., 2012; Schwenk et al., 2012; Wessels et al., 2013; de Almeida et al., 2016; Müller et al., 2016; Schimo et al., 2017; Senkler et al., 2017; Vidoni et al., 2017; Rugen et al., 2019; Páleníková et al., 2021b).

Other separation techniques, such as clear native PAGE, sucrose gradient centrifugation, and size exclusion chromatography, have been successfully used to partition complexes by size and shape (Andersen et al., 2003; Peltier et al., 2006; Hartman et al., 2007; Chen and Williamson, 2013; Kirkwood et al., 2013; Larance et al., 2016; Páleníková et al., 2021a). However, the majority of complexomic studies now rely on a combination of orthogonal fractionation methods, thus decreasing the probability of chance co-elution for proteins involved in closely migrating but otherwise unrelated assemblies. Such fractionation schemes can be quite complex, include both parallel and sequential steps, and yield hundreds-to-thousands of fractions (Dong et al., 2008; Menon et al., 2009; Havugimana et al., 2012; Gordon et al., 2013; Wan et al., 2015; Gazestani et al., 2016; Shatsky et al., 2016). As a result, researchers have access to a wider variety of physical information about each complex and, based on richer complexome profiling datasets, can predict with higher confidence the involvement of each protein in macromolecular assemblies.

In one stunning methodological development, the sucrose gradient fractionation of the cell lysate is coupled to both mass spectrometry and cryo-electron microscopy—to directly classify and visualize complexes present in select fractions, solve their structures, and thereby identify the constituent proteins (Figure 2B). Pioneered by studies of bacterial ribosome assembly (Sashital et al., 2014; Davis et al., 2016), the technique has been recently extended to cover virtually any macromolecular complex in the cell (Ho et al., 2020). With this bottom-up endogenous structural proteomics approach, complexomics achieves its most visual expression and promises a wealth of exciting data in the years to come.

**FIGURE 3:**
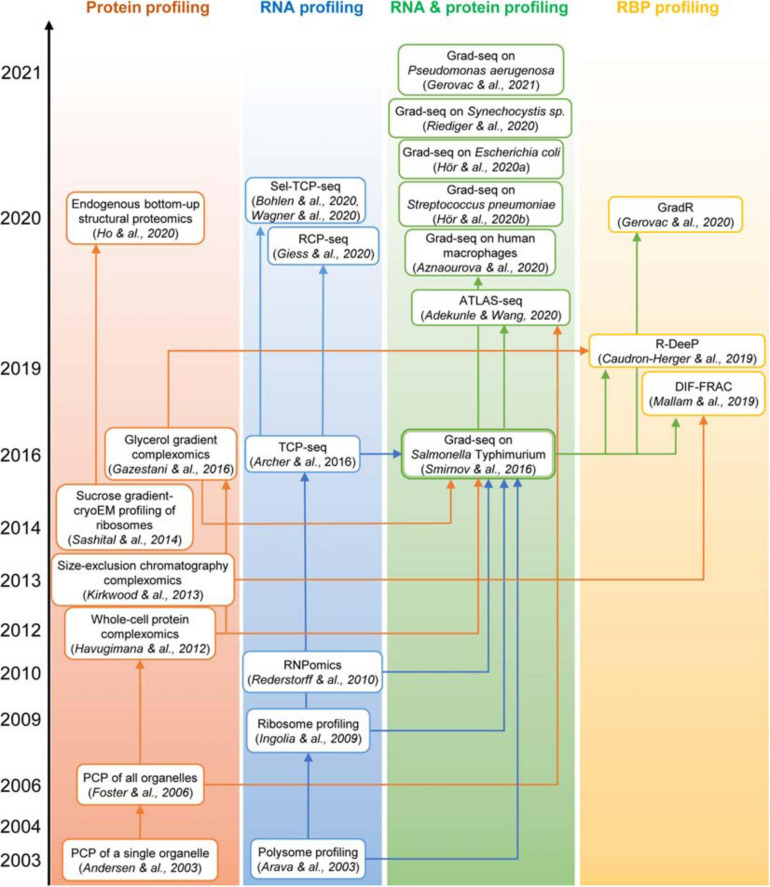
The “family tree” of principal complexomic approaches and the conceptual links between them. High-throughput techniques initially interrogating only the protein or only the RNA dimension of stable macromolecular complexes (the first two columns) inspired the development of the Grad-seq family of methods that profile both components simultaneously to infer stable RNA–protein associations (the third column). Adaptations of the latter technique have recently enabled the systematic identification of proteins involved in stable RNPs, be they genuine RBPs or indirectly associated subunits (the last column).

Because complexomic techniques do not require genetic manipulation of the biological system and provide a large amount of data on the physical organization of the proteome, they have been used to map complexomes not only of model but also of some traditionally “difficult” organisms, including numerous bacteria, archaea, protists, algae, plants, animals, and human subjects (Peltier et al., 2006; Kristensen et al., 2012; Chatzispyrou et al., 2017; Moutaoufik et al., 2019; Van Strien et al., 2019 and the references above). The biological insights brought about by these studies are impressive: from the organization of giant respiratory supercomplexes, through the fine structural coupling of metabolic pathways, to unexpected properties and compositions of gene expression machines, to new hints at possible functions of “orphan” proteins and molecular mechanisms of human diseases. From this “protein-only” complexomic perspective only few steps were remaining to the vast RNP world (Figure 3).

## Incorporating RNA in the Complexome Landscape

Though largely focused on proteins, some complexomic studies had already reserved a place of honor to major RNPs, most often ribosomes (Wessels et al., 2013; Gazestani et al., 2016; Larance et al., 2016; Chatzispyrou et al., 2017; Van Strien et al., 2019; Páleníková et al., 2021a). These works resulted in several interesting findings, including details of bacterial and mitochondrial ribosome assembly (Chen and Williamson, 2013; Sashital et al., 2014; Davis et al., 2016; Rugen et al., 2019) and some of the first observations of CRISPR ribonucleoproteins (Menon et al., 2009). However, because the RNA composition of fractions was not explicitly interrogated, the interpretation of a wide diversity of observed RBP distributions, e.g., those of mitochondrial PPR proteins (Wessels et al., 2013; Senkler et al., 2017; Rugen et al., 2019), numerous RNA-related complexes in chloroplasts (Peltier et al., 2006), or a potential RNA chaperone in archaea (Menon et al., 2009), was precluded.

First attempts to visualize the RNA component of RNPs relied on low-throughput northern blotting (e.g., Wassarman and Storz, 2000). The polysome and ribosome profiling, later joined by the translation complex profile sequencing (TCP-seq), the selective TCP-seq (Sel-TCP-seq) and the ribosome complex profiling (RCP-seq) (Figure 2C), marked a decisive turn in handling the RNA dimension by introducing the unbiased and comprehensive quantification of mRNAs isolated from defined sucrose gradient fractions by microarray and later RNA-seq (Arava et al., 2003; Ingolia et al., 2009; Archer et al., 2016; Bohlen et al., 2020; Giess et al., 2020; Wagner et al., 2020). Such approaches have revolutionized our view of dynamic translation but are naturally limited to specific types of class IIa (elongating or scanning ribosomes), class IIIc (stalled ribosomes), and sometimes lncRNA-ribosome complexes (van Heesch et al., 2014; Carlevaro-Fita et al., 2016). Another transcriptome-wide technique, RNPomics, that similarly resolves cellular complexes in a gradient, sequences the constituent RNAs, and focuses instead on the sedimentation range 10S–30S, thereby excluding ribosome-bound transcripts (Rederstorff et al., 2010). This procedure enriches well for RBP-bound noncoding RNAs, but because all RNP-containing fractions are ultimately pulled and analyzed together, it does not really profile them and thus cannot provide further information about the diversity and the composition of these RNPs. Such a feat has become a reality only with the advent of gradient profiling by sequencing and mass spectrometry (Grad-seq).

## Profiling Stable RNPs With Grad-Seq

### General Pipeline

Grad-seq was introduced in 2016 as a new technique to comprehensively chart the landscape of an organism’s stable RNA and protein complexes without resorting to tagging or enrichment of cellular components (Smirnov et al., 2016, 2017a). This hybrid method marries the logic of a traditional complexomic approach with the power of RNA-seq to quantitatively profile transcripts of all expressed genes (Figure 3). The Grad-seq pipeline (Figure 2D) begins with the native lysis of biological material (bacterial or human cell culture) at low temperature to stabilize dynamic complexes. The cleared lysate is then loaded onto a linear density gradient and subjected to velocity centrifugation to resolve cellular complexes across the size range of interest (typically up to monosomes). Upon centrifugation, the gradient is split in a series of equal-volume fractions, and their RNA and protein components are isolated. The distributions of selected macromolecules across the gradient can be analyzed with conventional methods, i.e., gel electrophoresis, staining, western and northern blotting. To profile proteins and transcripts genome-wide, each fraction is subjected to label-free LC-MS/MS and RNA-seq. The obtained data are normalized with the help of added spike-in molecules and the *bona fide* in-gradient distributions of individual macromolecules revealed with conventional methods. The result is a complete collection of sedimentation profiles of potentially all expressed proteins and RNAs present in the studied biological model.

Peaks in each distribution indicate the stable assemblies of profiled molecules. Calculation of the sedimentation coefficient for each fraction, based on the behavior of known complexes or standards, enables access to molecular weight estimates for any detected complex (Erickson, 2009). With this “sedimentation ruler” one can make reasonable assumptions regarding the complexity of each observed particle, e.g., evidence for oligomerization or the degree of heterogeneity. Besides this physical information, one can now compare profiles between them, and this is where the genuine power of the approach reveals itself. Like classical complexomics, Grad-seq permits to evaluate and either confirm or reject proposed stable associations between proteins and RNAs, based on the similarity or dissimilarity of their sedimentation profiles. When the profiles are compared with each other globally, e.g., with the help of such analytical tools as PCA and t-SNE, one can go even further and forward hypotheses about novel complexes between similarly distributed proteins and transcripts (Smirnov et al., 2016; Hör et al., 2020a,b).

Additionally, if uncharacterized transcripts or proteins co-segregate with other components, whose molecular role has been determined (e.g., RNAP, ribosomal subunits, RNA chaperones, or other established RBPs), one can leverage guilt-by-association and predict their involvement in a similar kind of processes or assemblies. Such hypotheses can be verified with the help of accessory techniques, such as RNA and protein pull-downs, to robustly establish the composition of the observed RNPs (Figure 2D). Therefore, Grad-seq bridges the biochemical profiling of cellular complexes with functional information, which makes it a versatile functional genomics approach.

### Case Study: Comparative Analysis of Bacterial Stable RNPs

**FIGURE 4:**
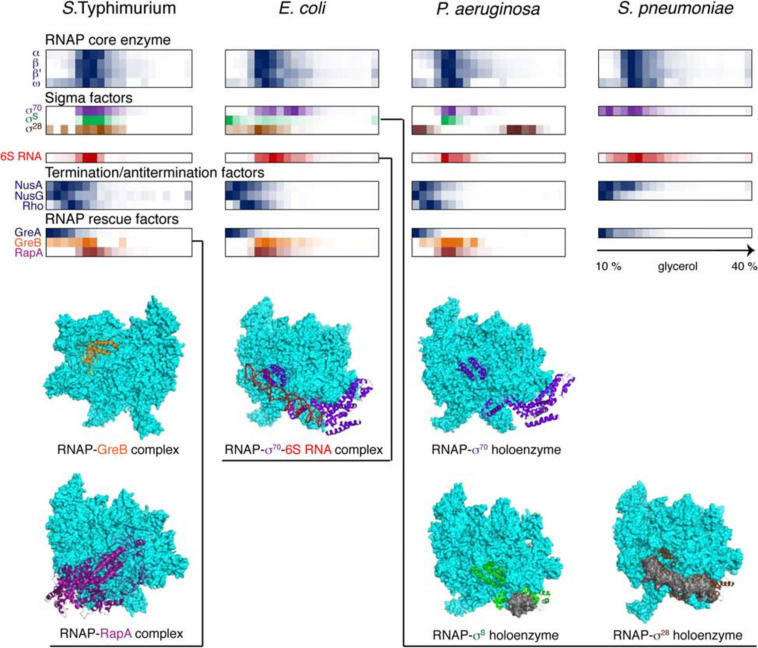
Back-to-back view of the transcriptional machinery from *S.* Typhimurium, *E. coli*, *P. aeruginosa*, and *S. pneumoniae* profiled by Grad-seq. The Grad-seq distributions of select proteins and RNAs, forming the RNAP holoenzyme or involved in transcription regulation and rescue, are shown as heat-maps reflecting the abundance of each macromolecule along the gradient. Some of the profiles highlighted with color are illustrated with the corresponding structures below (PDB codes: 6RIN, 4S20, 6P1K, 5IPL, 6PMI, and 5VT0). Note that *S. pneumoniae*, in contrast to the proteobacteria, has only one Gre factor.

**FIGURE 5:**
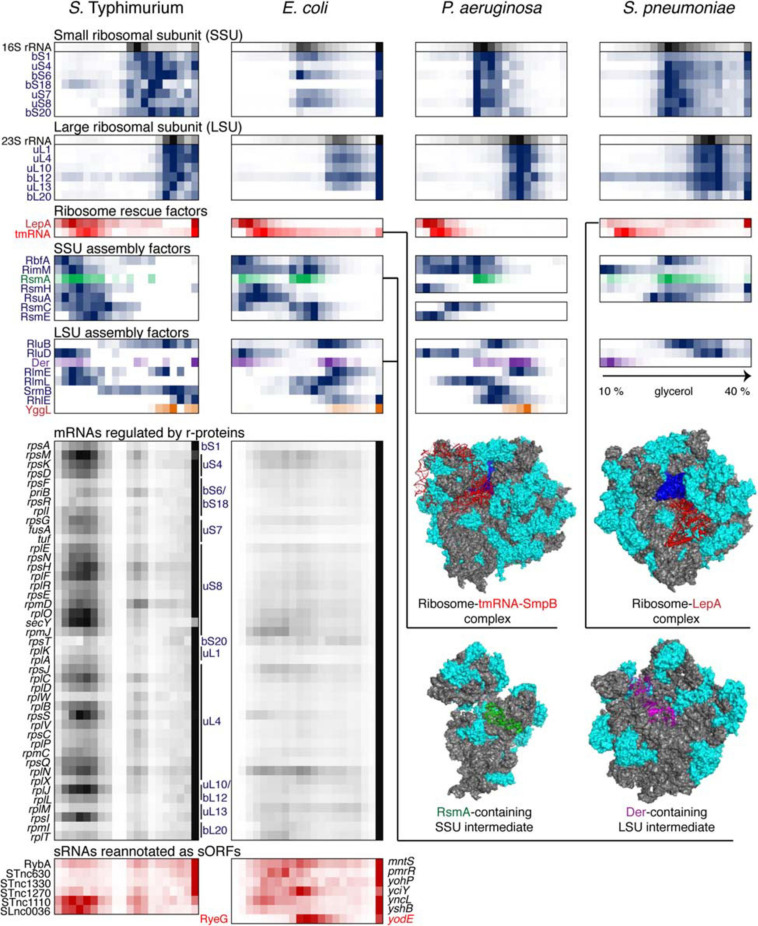
Back-to-back view of the translational machinery and the associated RNAs from *S.* Typhimurium, *E. coli*, *P. aeruginosa*, and *S. pneumoniae* profiled by Grad-seq. The Grad-seq distributions of select proteins and RNAs involved in the ribosome structure, assembly of rescue, are depicted in the same way as in Figure 4, and some of the complexes are illustrated with solved cryo-EM structures on the right (PDB codes: 6Q95, 5J8B, 4ADV, and 3J8G). Additionally, the Grad-seq profiles of autoregulated r-protein-encoding mRNAs are provided below to showcase their involvement in low molecular weight RNPs (left side of the profile) or association with translating ribosomes (last fraction). The r-proteins negatively regulating each cistron are specified on the right of the profiles. Certain enterobacterial sRNAs, shown in red at the bottom of the figure, have been re-annotated as short mRNAs due to the presence of a confirmed ORF and association with translating ribosomes. Their old sRNA identifiers are given on the left of each profile and the new approved protein-coding gene names are provided on the right.

**FIGURE 6:**
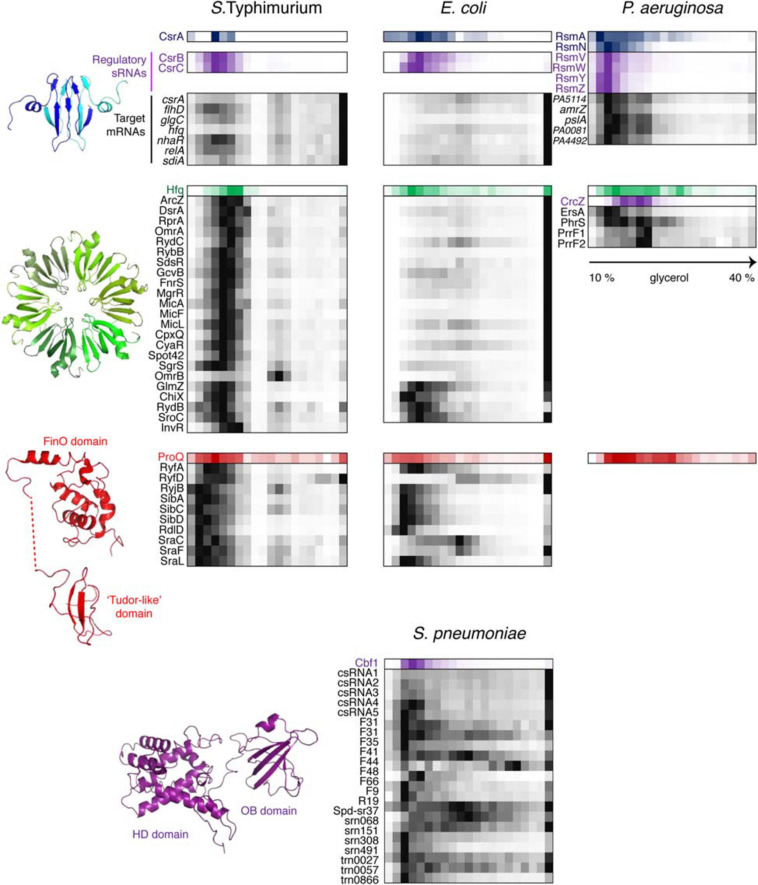
Back-to-back view of global regulatory RBPs and the associated RNAs from *S.* Typhimurium, *E. coli*, *P. aeruginosa*, and *S. pneumoniae* profiled by Grad-seq. The Grad-seq distributions of three major proteobacterial RBPs, CsrA, Hfq, and ProQ, and the *S. pneumoniae* exoribonuclease Cbf1 are shown along with select profiles of their sRNA and mRNA ligands (except for the *P. aeruginosa* ProQ, for which *in vivo* ligands have not been studied as for now). Decoy sRNAs in proteobacteria are highlighted with violet. The CsrA, Hfq, and ProQ structures—PDB codes: 1Y00, 2YLB, 5NB9, and 5NBB. The 3D structure of Cbf1 is predicted by RaptorX (Xu, 2019). The *P. aeruginosa* ProQ protein lacks the “Tudor-like” domain (Gerovac et al., 2021).

To date, Grad-seq has been used to profile stable RNPs in several model bacteria, such as the γ-proteobacteria *S.* Typhimurium (Smirnov et al., 2016; Gerovac et al., 2020; Venturini et al., 2020), *Escherichia coli* (Hör et al., 2020a), and *Pseudomonas aeruginosa* (Gerovac et al., 2021), the firmicute *Streptococcus pneumonia*e (Hör et al., 2020b), the cyanobacterium *Synechocystis sp.* (Riediger et al., 2020), and even in human cells (Aznaourova et al., 2020). Additionally, the RNPs formed by *P. aeruginosa* RNAs have been analyzed along with phage ΦKZ transcripts during viral infection in what can be considered the first “dual Grad-seq” experiment (Gerovac et al., 2021). These known biological models provided the first touchstone and an essential benchmark for this new technique. Figures 4–6 show back-to-back select examples of stable RNPs detected in the three proteobacteria and the evolutionarily distant *S. pneumonia*e. Let us consider them in further detail to see what kind of functional insights can be gleaned from such analysis.

The core transcriptional machinery (Figure 4) is well assembled in all examined species. RNAP is abundantly present as the holoenzyme, as can be judged by the cosedimentation of σ-factors with the core subunits in the 15-18S range, corresponding to transcription initiation or early elongation events. However, some elongation accidents have obviously taken place in the proteobacterial species, as can be attested by the recruitment of the elongation factor GreB, stimulating RNA cleavage and reactivation of deeply backtracked RNAP (Abdelkareem et al., 2019), and the ATPase RapA, forcing the translocation of stalled RNAP (Liu et al., 2015). Interestingly, GreA, specialized on very small backtracks usually caused by nucleotide misincorporation, is not recruited, highlighting the division of labor between the two paralogues present in enterobacteria (Borukhov et al., 1993). Similarly, the termination factors NusA, NusG, and Rho are at best only marginally associated with RNAP; indeed, Rho shows a peak of ∼10S, which agrees well with the 10.4S reported for a free hexamer (Geiselmann et al., 1992).

The translational machinery (Figure 5) features well-defined 30 and 50S subunits recognizable by their rRNA and r-protein markers. At the bottom of the gradient, 70S ribosomes engaged in translation are prominently represented; this is where most mRNAs naturally peak. Interestingly, endogenous *P. aeruginosa* mRNAs were observed mostly in low-molecular-weight complexes under these conditions. By contrast, during phage infection, viral messengers accumulated in ribosome-rich fractions, suggesting that they were effectively overtaking the translational apparatus of the cell (Gerovac et al., 2021).

Like transcription, translation elongation sometimes seems to go awry. In *S. pneumoniae*, the LepA GTPase, also known as elongation factor 4, is recruited to the ribosomes, suggesting inefficient translocation (Heller et al., 2017). In *E. coli*, the presence in the same fraction of tmRNA (in addition to the free tmRNA-SmpB complex sedimenting at 11–12S) betrays the accumulation of no-stop or no-go translation elongation complexes awaiting resolution (Keiler, 2015). In *S.* Typhimurium, both these aberrant states are clearly present. Similar to earlier observations (Chen and Williamson, 2013), several ribosome assembly intermediates appear to be abundant and long-lived enough to permit their detection by Grad-seq. These include the broadly conserved RsmA/KsgA and RbfA stages of 30S biogenesis, readily observable in all four bacteria (Datta et al., 2007; Boehringer et al., 2012). Association of other assembly factors with immature ribosomal subunits shows a great degree of species- and likely condition-specificity.

In this regard, it is appealing to compare in more detail the two enterobacterial datasets, given that *S.* Typhimurium and *E. coli* are genetically extremely close, and both were harvested during the transition between the exponential and the stationary phases of growth (Smirnov et al., 2016; Hör et al., 2020a). In both species, 6S RNA is already beginning to engage the housekeeping (σ^70^) form of RNAP, and the recruitment of σ^28^ marks the commitment of both cultures to foster motility at the wake of the exponential phase (Barembruch and Hengge, 2007). However, the *E. coli* σ^*S*^, unlike its *Salmonella* counterpart, does not yet have a privileged access to the transcriptional machinery (Figure 4). Moreover, most ribosomal proteins and mRNAs in *E. coli* sharply peak at the bottom of the gradient, indicating active translation (Figure 5). Considerably higher amounts of ribosome assembly intermediates are visible in *E. coli*, as compared to the *S.* Typhimurium culture, suggesting actively ongoing ribosome biogenesis (Chen and Williamson, 2013). In *S.* Typhimurium, but not *E. coli*, r-protein-encoding mRNAs appear to be significantly involved in low-molecular-weight complexes reminiscent of the already mentioned autoregulatory class IIIa RNPs (Meyer, 2018). The corresponding r-proteins are also more abundant in extraribosomal fractions in *S.* Typhimurium, suggesting that they may indeed engage in inhibitory complexes with their own messengers, as would be expected when the ribosome production is curtailed (Figure 5). Altogether, these observations suggest that, at the moment of harvest, the *E. coli* population was physiologically considerably more exponential than the *S.* Typhimurium one, which had already begun to show some stationary-phase hallmarks. This little exercise illustrates how analysis of stable complexes by Grad-seq can inform on the functional state of the studied system.

This state is to a large extent governed by class III and IV RNPs formed by key posttranscriptional regulators CsrA and Hfq which had their fair share in the three proteobacterial gradients (Figure 6). CsrA homologues are expectedly involved in class IIIb complexes with their sRNA decoys (Babitzke and Romeo, 2007). However, in *S.* Typhimurium and *P. aeruginosa*, a significant proportion of CsrA/Rsm proteins appears to have reached some of their mRNA targets, such as *flhDC*, *glgC* and *nhaR* in enterobacteria (Baker et al., 2002; Pannuri et al., 2012; Yakhnin et al., 2013) and *pslA*, *PA0081*, and *PA4492* in *P. aeruginosa* (Brencic and Lory, 2009; Irie et al., 2010). The majority of Hfq-dependent sRNAs in *S.* Typhimurium form complexes of ∼350 kDa, and even larger assemblies are visible in *P. aeruginosa*. Given the ∼67 kDa of one Hfq hexamer plus an average of ∼40 kDa sRNA, this suggests the existence *in vivo* of rather more complex RNPs than traditionally believed (Dimastrogiovanni et al., 2014; Obregon et al., 2015; Bandyra et al., 2016; Caillet et al., 2019). Interestingly, several *Salmonella* Hfq-dependent sRNAs, including ArcZ, DsrA, and RprA, co-migrate with RNAP, which is reminiscent of their antitermination activity during transcription of the σ^*S*^-encoding *rpoS* mRNA as cells enter the stationary phase (Sedlyarova et al., 2016). Remarkably, only part of Hfq-dependent sRNAs cosediment with the low-molecular-weight pool of Hfq in *E. coli*, whereas the majority are found in the pellet associated with translating ribosomes (Hör et al., 2020a). It appears that in this case most Hfq-dependent sRNAs follow their mRNA targets, while the free Hfq pool is usurped by a few high-affinity binders that are less inclined to interact with actively translated messengers.

The quick look at what Grad-seq permitted to reveal about known RNA-protein complexes inspired confidence in its ability to go beyond this canonical purview and interrogate new biology that had eluded the attention of researchers. Unexpected discoveries were not slow to appear.

### New RNPs Discovered by Grad-Seq

#### Class IIa: Translated Small ORFs in Presumed sRNAs

A common problem in the automatic *de novo* annotation of protein-coding genes is the default cut-off of 100 amino acids below which the genome sequence is typically not queried to avoid false positives (Harrison et al., 2002). This means that, in the absence of additional experimental evidence, key proteins such as CsrA (61 aa), RpoZ (91 aa), and many ribosomal proteins would have been simply missed—rather a disconcerting prospect. Molding the annotation from a related species by homology search improves the situation but does not sort the issue entirely out, since preexisting knowledge is still required, and certain ORFs—albeit functional—are too short and insufficiently conserved to be robustly detected (Storz et al., 2014). Therefore, direct experimental evidence of the polypeptide production is necessary. This can be achieved either by the identification of the molecule in question (by MS or western blotting) or by demonstrating the involvement of the corresponding mRNA in translation (Makarewich and Olson, 2017). The former approach has been recently used to support the existence of 170 small proteins, including 89 uncharacterized ones, in a *S.* Typhimurium Grad-seq dataset (Venturini et al., 2020). Their detection has become possible thanks to a higher sensitivity offered by Grad-seq protein profiling which, by fractionating the cell lysate, considerably decreases the complexity of analyzed samples. Moreover, 82 of the uncharacterized small proteins were found to form complexes with other molecules, providing first evidence of their functionality.

The first Grad-seq analysis unveiled the fundamental biochemical dichotomy between coding and noncoding RNAs in *Salmonella*: the former expectedly tended to cosediment with 30S and 70S ribosomes whereas the latter preferred small RNPs (Smirnov et al., 2016). The flagrant association of a few sRNAs with ribosomal particles urged to reconsider their coding potential and, with support of complementary experimental and comparative genomic evidence, re-annotate them as small mRNAs (Figure 5) (Hemm et al., 2008, 2010; Waters et al., 2011; Kato et al., 2012; Smirnov et al., 2016; Hör et al., 2020a). For example, the *E. coli* Grad-seq dataset exposed the interesting case of the prophage-encoded sRNA RyeG strongly associated with 30S and 70S ribosomes (Bak et al., 2015; Hör et al., 2020a). The existence within this transcript of a short *yodE* ORF, encoding a 48-aa bacteriostatic peptide, has been corroborated *in vitro* by 30S toeprinting and *in vivo* by modified ribosome profiling and functional assays, indicating that RyeG is in fact a minute mRNA (Weaver et al., 2019; Hör et al., 2020a).

#### Class IIb: New Ribosome Assembly Factors

Earlier studies showed that many proteins involved in ribosome biogenesis stably associate with free 30S and 50S subunits during gradient centrifugation, which can be exploited to predict new ribosome assembly factors (Chen and Williamson, 2013). The bacterial Grad-seq datasets revealed a few proteins cosedimenting with either subunit, including several uncharacterized ones (Smirnov et al., 2016; Hör et al., 2020a,b; Gerovac et al., 2021). One of them, the 12-kDa DUF469 protein YggL found in many γ- and β-proteobacteria, showed pronounced association with 50S and 70S particles (Figure 5). Given that *yggL* is mostly expressed in the exponential phase, and its deletion has a strong effect on the 50S levels, the protein likely functions in late LSU assembly (Chen and Williamson, 2013; Smirnov et al., 2016; Hör et al., 2020a). This specific example provides a *modus operandi* to study other candidate ribosome biogenesis factors spotted by Grad-seq, especially in less studied species (Hör et al., 2020b; Riediger et al., 2020).

#### Class IIIb: A Scaffolding lncRNA to Organize the Immune Response to Bacterial Infection

Grad-seq has been recently adapted to eukaryotic cells to study lncRNPs produced by immune cells in response to bacterial infection (Aznaourova et al., 2020). Human macrophages, activated by *Salmonella* lipopolysaccharide, were lysed and resolved on a glycerol gradient to comprehensively profile subribosomal RNPs. Focusing on the transcripts upregulated by infection-relevant stimuli, the authors noticed that the uncharacterized cytoplasmic lncRNA MaIL1 cosedimented with components of the ubiquitin-proteasome system. Its main partner, identified by RNA affinity purification and confirmed by Grad-seq profiling, is the ubiquitin-adapter optineurin (OPTN), which platforms the TBK1 kinase within the TLR4-TRIF pathway to induce type I interferon expression (Gleason et al., 2011; Munitic et al., 2013). MaIL1 is strictly required for the stability and the ubiquitin-dependent aggregation of OPTN. The emerging model posits that MaIL1 scaffolds the assembly of the ubiquitin-associated OPTN platform to enable type I interferon production and thereby activate antibacterial defense mechanisms (Aznaourova et al., 2020). A large repertoire of distinct lncRNPs revealed by Grad-seq in this study promises further surprises in this dynamically evolving research area (Walther and Schulte, 2020).

#### Class IIIc: A Trapped RNase to Protect sRNAs?

While sRNAs are omnipresent in bacteria, their associated proteins have much patchier phylogenetic distributions, and ample evidence indicates that, for instance, Hfq, important as it is in proteobacteria, does not play as pervasive a role in other bacterial clades (Zheng et al., 2016; Santiago-Frangos et al., 2017). Defining the organizational principles of regulatory sRNPs in Gram-positive bacteria has become a big challenge in microbial RNA biology over the last years. Consequently, performing Grad-seq in the model Firmicute *Streptococcus pneumoniae*, lacking all presently known global sRNA-binding RBPs, promised to be an exciting endeavor (Hör et al., 2020b). Gratifyingly, the 141 currently annotated pneumococcal sRNAs showed massive involvement in small RNPs of varying complexity. Some of them showed remarkably similar sedimentation patterns, suggesting shared RNP architecture (Figure 6). Capturing their protein partners from the cell lysate identified several candidate RBPs. One of them, the conserved 3′–5′ exoribonuclease Cbf1/YhaM (Oussenko et al., 2002; Lécrivain et al., 2018), strongly interacted with the five csRNAs, involved in competence control (Schnorpfeil et al., 2013; Laux et al., 2015), but not with differently distributed control transcripts. UV CLIP-seq corroborated this finding and revealed a number of additional sRNA and mRNA ligands, indicating that Cbf1 is in fact a global RNA binder. Importantly, the in-gradient distributions of many Cbf1-associated sRNAs turned out to be highly correlated with the Cbf1 profile, strongly supporting their involvement in the same stable sRNPs (Figure 6). The deletion of this unusual RNase, composed of an HD-domain (Histidine/Aspartate-containing metal-dependent phosphohydrolase) and an Oligonucleotide-Binding (OB)-fold, resulted in csRNA destabilization, supporting its protective role, similar to Hfq in Gram-negative bacteria (Oussenko et al., 2002; Hör et al., 2020b).

How can an RNase stably associate with—and even protect—RNA instead of destroying it? Cbf1 crosslinks tend to occur on the 3′-ends of transcripts, where this exoribonuclease normally cleaves (Hör et al., 2020b). However, the role of Cbf1 in cellular RNA turnover is at best modest (Oussenko et al., 2005; Lécrivain et al., 2018). In fact, *in vitro* assays and recent *in vivo* data revealed that Cbf1 trims the single-stranded 3′-oligo(U)-tail of its ligands by ∼3 nucleotides but fails to digest any further (Lécrivain et al., 2018; Hör et al., 2020b). The enzyme does not seem to be halted by a stable secondary structure, as no such feature has been observed around trimmed sites *in vivo* (Lécrivain et al., 2018), nor impeded by another RBP sitting on the 3′-end of the target RNA, since the same behavior has been recapitulated in a minimal system on protein-free RNA (Hör et al., 2020b). This lack of processivity may be an intrinsic feature of Cbf1 itself, which is reminiscent of the behavior of another HD-domain protein acting on the RNA 3′-end, the CCA-adding enzyme (Cca). Unlike template-dependent polymerases, Cca stably anchors to its tRNA substrate and does not translocate upon addition of nucleotides, which makes the catalyzed reaction self-limiting—it necessarily stops after addition of the 3-nucleotide CCA-tail (Shi et al., 1998; Cho et al., 2006; Kuhn et al., 2015). Whatever the exact mechanics of the Cbf1 cleavage is, current biochemical and functional evidence converges on a model where this exoribonuclease forms class IIIc RNPs (inhibited enzyme-substrate complexes) with its RNA ligands and thereby shields them from cellular degradative enzymes (Hör et al., 2020b).

Cbf1 and the associated csRNAs are involved in competence control in *S. pneumoniae* by repressing the *comC* mRNA (Schnorpfeil et al., 2013; Laux et al., 2015; Aprianto et al., 2018; Hör et al., 2020b). However, the large number of identified Cbf1 ligands and phenotypic data suggest that its physiological roles may go way beyond this small regulon (van Opijnen and Camilli, 2012; Lécrivain et al., 2018; Hör et al., 2020b). The other pneumococcal sRNPs uncovered by Grad-seq also await detailed characterization, promising identification of additional RBPs involved in riboregulation in Gram-positive bacteria (Zheng et al., 2017).

#### Classes IIIa and IV: ProQ, a New Global RNA Chaperone Acting on mRNAs and sRNAs

When Grad-seq entered the stage (Smirnov et al., 2016), CsrA and Hfq largely dominated the panorama of posttranscriptional regulation in enterobacteria (Van Assche et al., 2015). However, Grad-seq analysis of the RNPs formed by *Salmonella* sRNAs uncovered a surprising heterogeneity of profiles: while Hfq-binding sRNAs constituted a well-defined biochemical class of complexes (Figure 6), they accounted for only ∼30% of sRNA distributions, the other two thirds of noncoding RNAs being involved in different kinds of assemblies (Smirnov et al., 2016, 2017a). A group of such unusual transcripts clearly clustered together and were selected for RNA pull-downs to identify their shared protein partners. This analysis zeroed in on ProQ, an RNA chaperone of unknown functional role involved in osmoregulation (Milner and Wood, 1989; Kunte et al., 1999; Chaulk et al., 2011).

ProQ RIP-seq, later supported by CLIP-seq experiments, identified the same sRNAs and several hundred other transcripts, both coding and noncoding, as specific ProQ ligands (Smirnov et al., 2016; Holmqvist et al., 2018a). The ProQ-associated sRNAs are mostly Hfq-independent and, unlike classical Hfq-binding riboregulators, highly structured. Their in-gradient profiles correlated well with that of ProQ, indicating the formation of small (100-150 kDa) stable RNPs (Figure 6). The abundance and the stability of most ProQ-binding sRNAs and at least some mRNAs depend on ProQ which, similar to Hfq, tends to bind its ligands close to the 3′-end, protecting them from cellular RNases (Smirnov et al., 2016; Holmqvist et al., 2018a). RNA-seq analyses of Δ*proQ* strains uncovered a profound impact of this prolific RNA binder on gene expression in enterobacteria, making it, along with Hfq and CsrA, the third global regulatory RBP in this group (Smirnov et al., 2016; Melamed et al., 2020).

The ProQ research is now booming: from an obscure protein (unfairly) commanding only passing interest it has grown into a major topic of bacterial RNA biology (Olejniczak and Storz, 2017; Holmqvist et al., 2020). ProQ homologues have been found in a number of proteobacteria (Attaiech et al., 2016; Smirnov et al., 2016) and implicated in key physiological processes, including competence (Sexton and Vogel, 2004; Attaiech et al., 2016; Durieux et al., 2019), virulence, chemotaxis, motility and biofilm formation (Sheidy and Zielke, 2013; Westermann et al., 2019), metabolism (Kunte et al., 1999; Smirnov et al., 2016), stress resistance, plasmid and core genome maintenance (Smirnov et al., 2016; Bauriedl et al., 2020; Gerovac et al., 2020). Many known base-pairing sRNAs repressing their targets in-*cis* or in-*trans* turned out to be avid ProQ binders, but only in a handful of cases the role of this protein in regulation has been solved mechanistically (Smirnov et al., 2017b; Silva et al., 2019; Westermann et al., 2019), and an even larger number of ProQ-dependent sRNAs remain totally uncharacterized. Pioneering biochemical studies highlighted generic RNA chaperone properties of ProQ homologs (Chaulk et al., 2010, 2011). Their role as new RNA matchmakers, mechanistically distinct from Hfq, is being intensively studied, but the molecular details of the interplay between ProQ and its natural RNA ligands only begin to emerge (Melamed et al., 2020). Structural and functional studies highlighted the importance of the conserved N-terminal FinO-like domain, which harbors most of the RNA-binding activity and places ProQ into a larger family of FinO-like RNA chaperones (Chaulk et al., 2010, 2011; Glover et al., 2015; Gonzalez et al., 2017b; Eidelpes et al., 2020; Gerovac et al., 2020, 2021; Immer et al., 2020; Pandey et al., 2020; Stein et al., 2020). However, the role of the C-terminal “Tudor-like” domain (Figure 6), that contributes to the RNA chaperone activities of ProQ (Chaulk et al., 2011; Gonzalez et al., 2017b), does not cease to intrigue researchers. Recent comparative analysis uncovered striking structural and functional similarities between several small β-barrel (SSB) folds, such as Sm, OB, cold-shock and Tudor domains, and proposed a unifying concept of “urfold” highlighting their biological relatedness (Youkharibache et al., 2019). The remarkable frequency of the SSB urfold among bacterial global RBPs, including Hfq, ribosomal protein S1, Cbf1, ProQ, PNPase, RNases E and R, suggests that it may constitute a recurrent signature of pleiotropic RNA-binding regulators.

## Beyond Grad-Seq

The basic Grad-seq pipeline has recently seen two interesting adaptations, marking new important developments in RNA-protein complexomics (Figure 3). In one case, ATLAS-seq brings the complex profiling logic to the cell biology level: entire organelles from mildly lysed cells are resolved on a sucrose gradient and systematically profiled for their protein and RNA contents (Foster et al., 2006; Adekunle and Wang, 2020). Applied to mouse liver, this method revealed genome-wide distributions of transcripts across diverse cellular microenvironments, identified cases of differential localization for mRNA isoforms, and visualized RBPs following their targets (Adekunle and Wang, 2020).

Since Grad-seq studies broadly illustrated the dependence of RBP sedimentation on their RNA ligands (Figures 4–6), three independently developed approaches have leveraged this principle to systematically profile the protein components of stable RNPs (Caudron-Herger et al., 2019; Mallam et al., 2019; Gerovac et al., 2020). They do so by comparing global MS-derived protein profiles in lysates treated or not with an RNase, as destruction of the RNA component typically shifts the distribution of the associated proteins to lighter fractions or causes their disassembly. Thus, R-Deep employed the sucrose gradient fractionation of HeLa S3 cells and identified 1,784 proteins whose distributions changed upon RNase treatment, of which only ∼70% were known to be direct RNA binders (Caudron-Herger et al., 2019). Similarly, DIF-FRAC resolved complexes by size-exclusion chromatography and found >1,000 RNA-dependent proteins in HEK293T and mouse embryonic stem cells, of which ∼40% lacked RBP annotation. Based on the effect of the RNase treatment on the RNP behavior, all RNA-protein complexes were broadly classified into apo-stable (i.e., persisting even in the absence of RNA), structural (fully dependent on the RNA component), and compositional (with only some protein subunits contingent on the presence of RNA) (Mallam et al., 2019). Finally, GradR profiled *Salmonella* protein complexes on glycerol gradients and revealed a number of RNA-dependent proteins, including Hfq, CsrA, and ProQ, while also spotting an uncharacterized plasmid-encoded FinO/ProQ homolog, FopA, as a potential RBP. RIP-seq and *in vitro* assays confirmed its specific binding to the Inc sRNA and showed that FopA dramatically accelerates the base-pairing between Inc and the *cis*-encoded *repZ* mRNA involved in plasmid replication (Gerovac et al., 2020). This study brought to three the number of ProQ/FinO-like chaperones simultaneously residing in the same bacterial cell (ProQ, FinO, FopA), which is an exceptional case with potentially interesting evolutionary implications. All in all, these approaches beautifully complement the existing RBP discovery techniques, such as RNA interactome capture and the new crosslinking-based methods OOPS, PTex, TRAPP, and XRNAX (Hentze et al., 2018; Queiroz et al., 2019; Shchepachev et al., 2019; Trendel et al., 2019; Urdaneta et al., 2019; Smith et al., 2020b), by adding those RNP components which do not interact with RNA directly or crosslink poorly yet contribute to the structure and the functionality of the complexes. Like the classical Grad-seq, they provide important biochemical data on the organization and the RNA dependency of cellular RNPs.

## Outlook

Considering Grad-seq warts-and-all, next to the advances it brought about in six model species, the method still must face up to a few challenging issues. The most important one is profile matching. A recent critical appraisal exposed a surprisingly high false discovery rate of global complexomic analyses, when complex membership is called by direct matching of protein distributions (Shatsky et al., 2016). This happens despite the ever more sophisticated bioinformatic pipelines used for the analysis of complexomic data (Dong et al., 2008; Havugimana et al., 2012; Kristensen et al., 2012; Giese et al., 2014; Gazestani et al., 2016; Páleníková et al., 2021a). The problem comes, on the one side, from natural spurious overlapping of unrelated profiles, and on the other, from the general analytical caveat of matching algorithms: most statistical measures are inherently tailored to look for differences, not similarities (Motulsky, 2010). For the moment, Grad-seq suffers from the same limitation: while clustering algorithms and especially dimensionality reduction methods (PCA, t-SNE) could be applied to classify and compare profiles, predicting complexes based solely on such profile similarities is dangerous. Therefore, Grad-seq prudently incorporates an RNA pull-down step to prioritize protein candidates and only then performs profile matching to call RNPs – expectedly with a much higher success rate (Figure 2D). It is desirable, however, to develop in future more elaborate similarity metrics and information-rich profile descriptors to enable automated detection of RNPs from Grad-seq datasets with acceptable accuracy and sensitivity.

Data mining is another issue. The Grad-seq publications highlighted in this review primarily reported on a few particularly striking findings illustrating the power of the method. But those are merely the tip of the iceberg, and the RNA and especially the protein Grad-seq datasets await deeper exploration to unearth new potentially interesting macromolecular assemblies. This is particularly important if we want to approach the functions of understudied proteins and transcripts.

We contemplate many possible avenues for further development of RNP complexomics. It will be exciting to apply Grad-seq family approaches to more exotic and recalcitrant biological systems, such as newly discovered microbes, evolutionarily diverged eukaryotes, and semi-autonomous organelles, like mitochondria and chloroplasts, in search for novel RNA biology (Castelle and Banfield, 2018; Adl et al., 2019). We foresee that RNP complexomics will become increasingly comparative, confronting different species or assessing how the ensemble of stable complexes within the same organism changes between conditions or in time. Experimental settings and statistical tools have definitely matured to carry out such complex studies (Heide et al., 2012; Kristensen et al., 2012; Moutaoufik et al., 2019; Van Strien et al., 2019; Gerovac et al., 2021; Páleníková et al., 2021a,b). Finally, albeit the identification of RNA from cryoEM structures remains challenging (Greber et al., 2014), the expansion of the bottom-up structural proteomics (Figure 2B, Ho et al., 2020) to the RNP world is now as tantalizing a perspective as never before.

## Author Contributions

AS designed the study, analyzed data, wrote the original draft, and prepared the figures. MG and JV contributed to the data and reviewed the manuscript. All authors contributed to the article and approved the submitted version.

## Conflict of Interest

The authors declare that the research was conducted in the absence of any commercial or financial relationships that could be construed as a potential conflict of interest.
